# Pollen grading prediction scale for patients with *Artemisia* pollen allergy in China: A 3‐day moving predictive model

**DOI:** 10.1002/clt2.12280

**Published:** 2023-07-10

**Authors:** Zhaoyin Yin, Yuhui Ouyang, Bing Dang, Luo Zhang

**Affiliations:** ^1^ Institute of Urban Meteorology China Meteorological Administration Beijing China; ^2^ Beijing Meteorological Service Center Beijing China; ^3^ Department of Allergy Beijing Tongren Hospital Capital Medical University Beijing China; ^4^ Beijing Key Laboratory of Nasal Diseases Beijing Institute of Otolaryngology Beijing China; ^5^ Research Unit of Diagnosis and Treatment of Chronic Nasal Diseases Chinese Academy of Medical Sciences Beijing China; ^6^ Beijing Municipal Climate Center Beijing China

**Keywords:** *Artemisia* pollen, Beijing, China, pollen allergy, pollen deposition graded prediction

## Abstract

**Background:**

*Artemisia* pollen is the most prevalent outdoor aeroallergen causing respiratory allergies in Beijing, China. Pollen allergen concentrations have a direct impact on the quality of life of those suffering from allergies. *Artemisia* pollen deposition grading predictions can provide early warning for the protection and treatment of patients as well as provide a scientific basis for allergen specific clinical immunotherapy.

**Objective:**

To develop a model of *Artemisia* pollen grading to predict development in patients with pollen allergy.

**Methods:**

*Artemisia* pollen data from four pollen monitoring stations in Beijing as well as the number of *Artemisia* pollen allergen serum specific immunoglobulin E positive cases in Beijing Tongren Hospital from 2014 to 2016 were used to develop a statistical model of pollen deposition and provide optimised threshold values.

**Results:**

A logarithmic correlation existed between the number of patients with *Artemisia* pollen allergy and *Artemisia* pollen deposition, and the average pollen deposition for three consecutive days was most correlated with the number of allergic patients. Based on the threshold of the number of patients and the characteristics of *Artemisia* pollen, a five‐stage pollen deposition grading model was developed to predict the degree of pollen allergy.

**Conclusions:**

Graded prediction of pollen deposition may help pollen allergic populations benefit from preventive interventions before onset.

## 1 BACKGROUND

While plants benefit human lives, they also produce pollen that may affect human health.^1^ This phenomenon is exacerbated by climate change and rising levels of carbon emissions,^2^, ^3^ which cause respiratory^4^ and digestive^5^ diseases. Artemisia pollens have been recognised as a major cause for late summer and autumn seasonal allergic respiratory disease worldwide, particularly in northern China, where 33.6%–58.2% allergy patients were sensitised to Artemisia pollen.^6^, ^7^ Respiratory allergies caused by Artemisia pollen have a considerable impact on patients' quality of life and comfort, and results in a substantial burden on the economy through health care costs and the reduction in productivity.^8^ Aeroallergen concentrations have a direct impact on the symptoms and quality of life of those suffering from allergies.^9^ Therefore, the establishment of a prediction model based on the correlation between pollen concentration level and patient symptoms may help to protect and treat patients.

Recent studies have used self‐examination report data^10^, ^11^ and provided grading thresholds. However, these studies mainly used volumetric samplers for pollen collection, while the results of analyses based on cost‐effective and accessible gravitational methods were rarely reported despite there being a correlation between the estimates obtained using these two methods.^12^ It is critical to develop grading criteria based on case data that can be used in developing countries where the number of pollen‐sensitive patients is rapidly increasing.^13^, ^14^, ^15^

In this study, we analysed the relationship between daily *Artemisia* pollen deposition and the number of patients with pollen allergies in Beijing from 2014 to 2016. We also aimed to establish a graded prediction model for pollen allergies.

## 2 METHODS

### 2.1 Pollen detection and counting

Pollen grains were collected continually for 24 h per day from 1 January 2014 to 31 December 2016 using a gravitational Durham sampler (Yamato Co. Ltd.). Grains were counted under a microscope with a counting unit of grain/1000 mm^2^. *Artemisia* pollen data were obtained from the meteorological observation fields of Chaoyang (39°57′N, 116°30′E), Fengtai (39°52′N, 116°15′E), Haidian (39°59′N, 116°17′E), and Shiijngshan (39°57′N, 116°12′E) District Meteorological Bureaux, all within the Beijing urban area, China (Supplementary Figure S1). The daily pollen deposition value in Beijing was noted as the average of the four sampling sites.

The start and end dates of the pollen season are usually determined based on the total amount of pollen, where the start and end amounts are 2.5% and 97.5% of the annual pollen amount, respectively.^16^

### 2.2 Patient data

Patient data were provided by the Beijing Tongren Hospital from 2014 to 2016 and represented the daily number of patients with both *Artemisia* pollen allergies and allergic symptoms such as sneezing, rhinocnesmus and runny nose. Pollen allergies were expressed as sIgE levels, measured using the Pharmacia UniCAP system (Thermo Fisher Scientific China Co., Ltd.).

### 2.3 Sample selection

The allergenicity of pollen depends on the biosynthesis before pollination. Pollen grains have developed a protective mechanism against the effects of radiation, heat and water loss while transported in the atmosphere, even after days in the atmosphere pollen grains still contain reactive allergens.^17^, ^18^ Therefore, settlement data obtained on a single day may not provide sufficient information. Thus, the daily pollen deposition was extended to a 2–4 days moving average deposition to increase data and reduce the influence of singular values (e.g., the 2‐day average pollen deposition represented the average pollen grains of the current and previous days).^19^, ^20^ Additionally, patient data obtained on holidays were excluded because of differences in the hospital capacity on holidays and weekdays. Ultimately, a total of 111 paired samples met the eligibility requirements.

### 2.4 Modelling

First, the daily number of patients and pollen deposition values were considered dependent and independent variables, respectively, and a Spearman correlation analysis was used to examine their association. High‐correlation data were selected as modelling samples. Second, the independent variable values were sorted in the ascending order to determine two series of data that were used as the basic thresholds: (1) pollen deposition values in the 25th, 50th, and 75th percentiles and (2) deposition values when 25%, 50%, and 75% of patients appeared. Third, a fitting function was constructed, and its first derivative was obtained to analyse the change of dependent variable with the independent variable in the interval formed by the basic threshold. Finally, the deposition values of 25th, 50th, and 75th percentiles were also introduced to the function to find possible points of mechanism significance which would be regarded as new thresholds, and where an optimised pollen deposition level could be established based on all threshold values.

## 3 RESULTS

### 3.1 Characterisation of the pollen deposition and the number of patients

**FIGURE 1:**
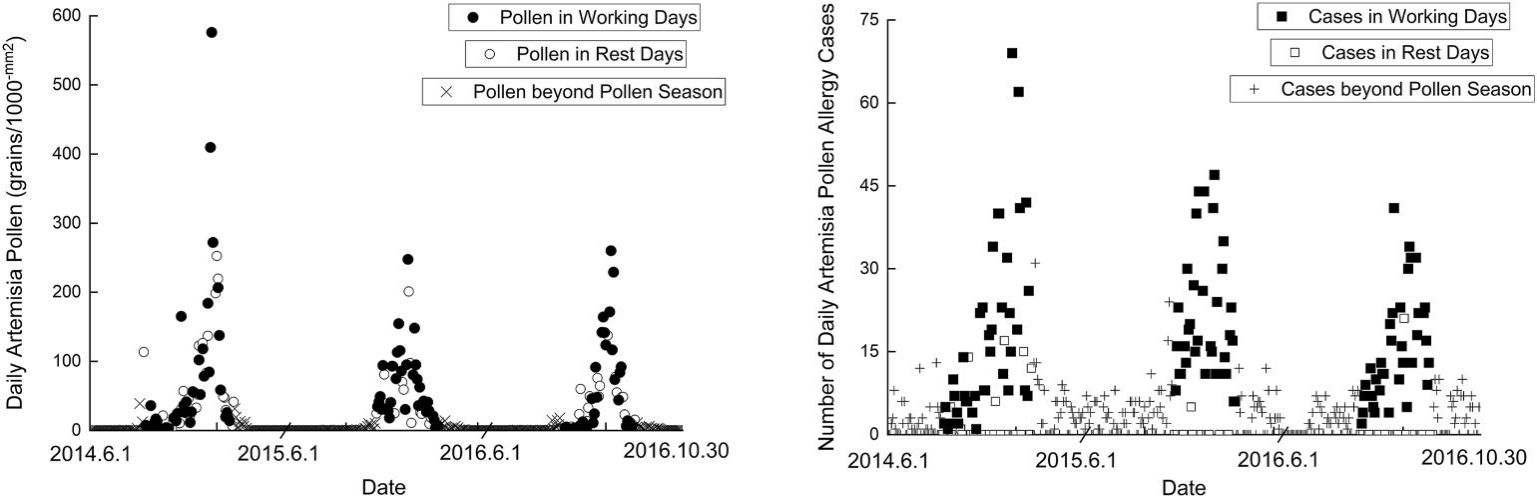
The average daily pollen deposition and the number of people allergic to *Artemisia* recorded in the Beijing urban area during 2014–2016. (Left) The average daily pollen deposition. Solid dots: pollen grain data. Hollow dots: pollen grain values on rest days. Crosses: pollen grain values outside the pollen season. (Right) The number of people allergic to *Artemisia*. Solid squares: allergy cases included in the analysis. Hollow squares: cases on rest days. Cross: Allergy cases recorded outside the pollen season.

The average daily *Artemisia* pollen deposition values and the corresponding number of patients with *Artemisia* pollen allergy in Beijing (2014–2016) are presented in Figure 1. During the study period, the *Artemisia* pollen season started on different days (12 July 2014, 9 August 2015, and 31 July 2016) and tended to end consistently in mid‐September. A total of 4845, 3017, and 3008 *Artemisia* pollen grains were collected with only 2935, 2106, and 1950 grains collected during the weekdays of the seasons of 2014, 2015, and 2016, respectively. Additionally, a total of 1,020, 1,080, and 795 patients with 694, 716, 546 patients in weekdays were found in the 2014, 2015, and 2016 pollen seasons, respectively.

### 3.2 Correlation analysis

The correlation coefficients for the number of patients and average pollen deposition in between 1 and 4 days were analysed, and we determined that the number of patients had the best correlation with 3‐day moving average values (Table 1). Therefore, only the effect of 3‐day moving average pollen depositions was considered for our model. Results of other independent variables are shown in Supplementary Figure S2.

**TABLE 1:** Spearman correlation between the number of patients with *Artemisia* pollen allergy and the daily, 2‐day, 3‐day and 4‐day moving average pollen deposition.

	Daily pollen deposition	2‐day average pollen deposition	3‐day average pollen deposition	4‐day average pollen deposition
Cases	0.569**	0.608**	0.609**	0.597**

**Correlation is significant at the 0.01 level.

### 3.3 Modelling

**FIGURE 2:**
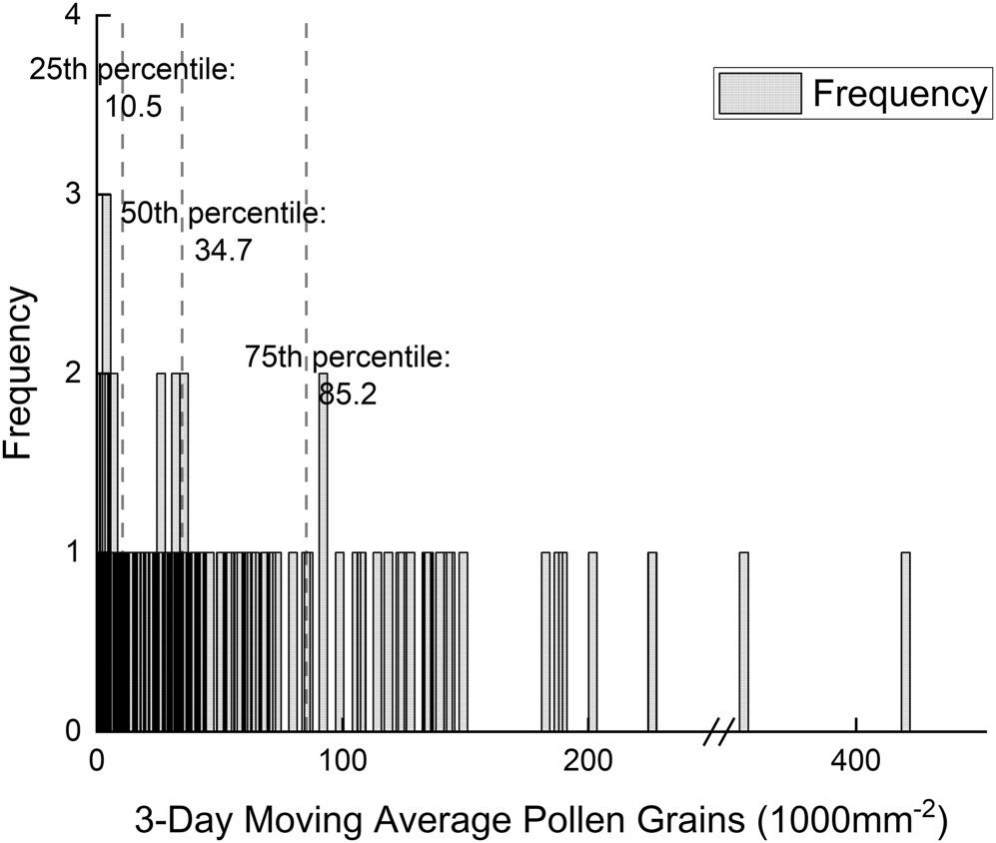
The frequency of the 3‐day moving average pollen deposition and its 25th, 50th, and 75th percentiles.

**FIGURE 3:**
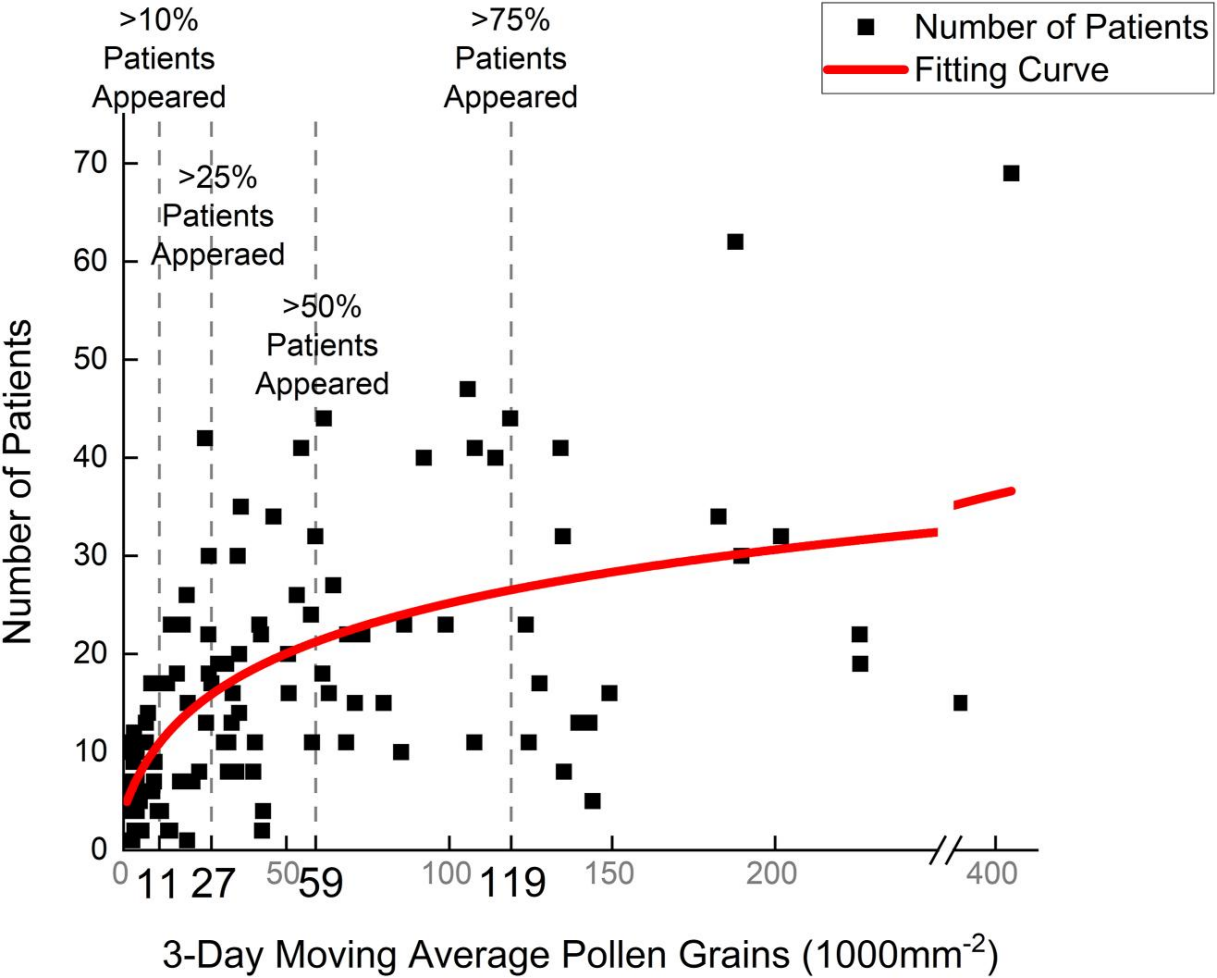
The association between the number of patients with *Artemisia* allergy and the 3‐day average pollen deposition.

The 3‐day moving average pollen deposition frequency in the 25th, 50th, and 75th percentile values were 10.5, 34.7, and 85.2 grains/1000 mm^2^ (Figure 2). The association between the number of patients with *Artemisia* allergy and the 3‐day average pollen deposition was analysed. The analogue curve is shown in Figure 3, and the equation of best fit is shown in Table 2.

**TABLE 2:** The fitting equation, first‐order derivative, and threshold criterion of the 3‐day moving average pollen deposition statistical model.

Independent variable	Fitting formula and *R* ^2^	First derivative	Criteria	Pollen deposition threshold (1000 mm^−2^ day^−1^)
3‐day moving average pollen deposition	f(x)=8.33×ln(x+8.53)−13.85	$f^{‘}\left(x\right)=8.33/\left(x+8.53\right)$	10% patients appeared	11
			25% patients appeared	27
	*R* ^2^ = 0.34			
			50% patients appeared	59
			75% patients appeared	119

According to the fitting curve, pollen deposition amounts corresponding to 25%, 50%, 75% of patients were set as grading thresholds. However, the initial minimum threshold values, which began after 25% of patients appeared, were suspected of not factoring in the early stages of the pollen season. Therefore, the 25th percentile of pollen deposition (11 grains/1000 mm^2^) value along with a corresponding 10% of patients was designated as the new minimum threshold. Based on these four threshold values, a 5‐grade optimised pollen deposition‐level scale was built (Table 2).

Furthermore, the increase in the number of patients and pollen deposition near the threshold points could be determined quantitatively by substituting the values into the first derivative equation. When pollen deposition reached 11, 27, 59, and 119 grains/1000 mm^2^, a one‐grain increment would then result in an increase in the number of patients by 0.42, 0.23, 0.12, and 0.06, respectively. Upper and lower limits for the increase in the number of patients at different levels are shown in Table 3.

**TABLE 3:** The optimised 3‐day moving average pollen deposition levels and the growth of patients with pollen deposition in each grade.

	Optimised pollen deposition level	Additional patients when 3‐day moving average pollen deposition increase 1
3‐day moving average pollen deposition	Level 1, pollen deposition ∈ (0, 11]	≥0.42
	Level 2, pollen deposition ∈ (11, 27]	[0.23, 0.42)
	Level 3, pollen deposition ∈ (27, 59]	[0.12, 0.23)
	Level 4, pollen deposition ∈ (59, 119]	[0.06, 0.12)
	Level 5, pollen deposition ∈ (119, ∞)	＜0.06

The results of the daily, 2‐day moving average, and 4‐day moving average pollen deposition were also calculated and are shown in Supplementary Figure S3 and Suppplementary Tables S1 and S2.

## 4 DISCUSSION

In this study, the correlation between pollen deposition and daily number of *Artemisia* sIgE‐positive patients was analysed and a prediction grading model of *Artemisia* pollen allergy was established.

From the correlation analysis and the equation of fit, the number of patients with *Artemisia* allergy was most correlated with a 3‐day moving average of *Artemisia* pollen deposition, but was not correlated with the daily pollen deposition. This result was consistent with previous studies.^17^ Pollen grains can maintain reactive allergens in the atmosphere even after days, potentially influenced by meteorological factors such as rainfall, wind, and humidity.

Our results also indicate that the number of patients with *Artemisia* allergy increased logarithmically with a 3‐day moving average *Artemisia* pollen deposition. When the 3‐day average *Artemisia* pollen deposition was between 11 and 27 grains/1000 mm^2^, an increase in the pollen deposition led to a rapid increase in the number of patients, with a one‐grain increment resulting in an increase in the number of patients by 0.42. However, when the 3‐day average *Artemisia* pollen deposition was more than 119 grains/1000 mm^2^, the increase in the number of patients slowed, with a one‐grain increment increase resulting in an increase in the number of patients by 0.06. Studies have also verified that the pollen deposition and allergy reaction association is not linear, unlike that in grass‐sensitised patients.^21^, ^22^ Patients are highly sensitised to *Artemisia* pollen allergens and often experience severe allergic reactions. Therefore, even a small dispersal amount of *Artemisia* pollen early in the season may induce symptoms and lead to a rapid increase in the number of patients. On the other hand, when pollen depositions are high, many patients may also use medications to relieve symptoms, resulting in a slow increase in patients.

Pollen deposition grading thresholds provide an effective time window for the prevention and treatment of allergic rhinitis. However, there are a variety of grading standards,^23^, ^24^ with some studies using subjective criteria^25^ or percentiles of pollen deposition,^26^ which may ignore patient statistics. In this study, pollen deposition values were graded in conjunction with the proportion of patients at 10%, 25%, 50%, and 75%, which increased the grading accuracy.

Many studies have also defined pollen stages^27^ or the threshold values^28^ based on symptom scores, but due to differences in geography, climate, and vegetation, these grading scales may not be applicable in China. In this study, a grading study was carried out based on both the number of patients and the characteristics of *Artemisia* pollen in Beijing; then, the growth of patient numbers at one‐grain increments in each level was calculated quantitatively. This showed good clinical significance, particularly for areas that have not yet implemented symptom self‐assessment gradings according to the number of patients.

However, due to limitations in observation instruments, this study only obtained the quantity of pollen deposition, rather than the pollen concentration in the unit of volume.

## 5 CONCLUSIONS

In conclusion, we found significant correlations between the number of *Artemisia* pollen sIgE‐positive patients and pollen deposition amounts. Additionally, we established a grading model for *Artemisia* pollen.

## AUTHOR CONTRIBUTIONS

**Zhaoyin Yin:** Data curation; formal analysis. **Yuhui Ouyang:** Conceptualisation; project administration; supervision; formal analysis. **Bing Dang:** Data curation; resources. **Luo Zhang:** Conceptualisation, methodology, project administration, supervision, formal analysis.

## CONFLICT OF INTEREST STATEMENT

The authors declare that they have no competing interests.

## Supporting information

Supporting Information S1

Figure S1

Figure S2

Figure S3

Figure S4

Figure S5

Figure S6

Figure S7

## Data Availability

All data generated or analysed during this study are included in this published article (and its supplementary information files).
