# Supplementary material for: Pollen grading prediction scale for patients with Artemisia pollen allergy in China: A 3‐day moving predictive model
Source: Clin Transl Allergy. 2023 Jul 10;13(7):e12280. doi: 10.1002/clt2.12280 (PMC10332133; doi:10.1002/clt2.12280)
Supplement: Supplementary file 1 — Supporting Information S1 [file CLT2-13-e12280-s008.docx]

**SUPPLYMENTAR FIGURES LEGENDS AND TABLES**

**Supplementary Figure 1.** Location of pollen sampling sites and that of the hospital that provided patients’ data.

**Supplementary Figure 2.** The frequency of the daily(a), 2-day(b) and 4-day(c) moving average pollen deposition and their 25th, 50th, 75th percentile.

**Supplementary Figure 3.** The association between the number of patients with *Artemisia* allergy and the daily, 2-day and 4-day moving average pollen deposition.

**Supplementary Table** **1**. The fitting equation, first-order derivative, and threshold criterion of the daily, 2-day, 4-day moving average pollen depositions’ statistical models

| Independent variable | Fitting formula and R^2^ | First derivative | Criteria | Pollen deposition threshold (1000mm^-2^·day^-1^) |
| --- | --- | --- | --- | --- |
| Daily pollen deposition | $f\left(x\right)=4.29\times\ln(x+2.15)+3.83$R^2^=0.26 | $f^{\text{‘}}\left(x\right)=4.29/(x+2.15)$ | 25th percentile deposition & 10% patients appeared  25% patients appeared  50% patients appeared  75% patients appeared | 6  26  56  117 |
| 2-day moving average pollen deposition | $f\left(x\right)=6.78\times\ln(x+4.96)-6.81$R^2^=0.32 | $f^{\text{‘}}\left(x\right)=6.78/\left(x+4.96\right)$ | 25th percentile deposition & 10% patients appeared  25% patients appeared  50% patients appeared  75% patients appeared | 10  24  58  122 |
| 4-day moving average pollen deposition | $f\left(x\right)=8.5\times\ln(x+8.23)-14.66$R^2^=0.32 | $f^{'}\left(x\right)=8.5/(x+8.23)$ | 25th percentile deposition & 10% patients appeared  25% patients appeared  50% patients appeared  75% patients appeared | 13  31  64  105 |

**Supplementary Table** 2. The optimized daily, 2-day and 4-day moving average pollen deposition levels and the growth of patients with pollen deposition in each grade.

|  | Optimized pollen deposition level | Additional patients when 3-day moving average pollen deposition increase 1 |
| --- | --- | --- |
| Daily pollen deposition | $\left\{\begin{matrix} Level 1, Pollen Deposition\in(0 , 6] \\ Level 2, Pollen Deposition\in(6 , 26] \\ Level 3, Pollen \text{Deposition}\in(26 , 57] \\ Level 4, Pollen Deposition\in(56 , 117] \\ Level 5, Pollen Deposition\in(117 , \infty) \end{matrix}\right.$ | ≥0.52  (0.15, 0.52]  (0.07, 0.15]  (0.03, 0.07]  ＜0.03 |
| 2-day moving average pollen deposition | $\left\{\begin{matrix} Level 1, Pollen Deposition\in(0 , 10] \\ Level 2, Pollen Deposition\in(10 , 24] \\ Level 3, Pollen \text{Deposition}\in(24 , 58] \\ Level 4, Pollen Deposition\in(58 , 122] \\ Level 5, Pollen Deposition\in(122 , \infty) \end{matrix}\right.$ | ≥0.45  (0.23, 0.45]  (0.1, 0.23]  (0.05, 0.1]  ＜0.05 |
| 4-day moving average pollen deposition | $\left\{\begin{matrix} Level 1, Pollen Deposition\in(0 , 13] \\ Level 2, Pollen Deposition\in(13 , 31] \\ Level 3, Pollen \text{Deposition}\in(31 , 64] \\ Level 4, Pollen Deposition\in(64 , 105] \\ Level 5, Pollen Deposition\in(105 , \infty) \end{matrix}\right.$ | ≥0.4  (0.21, 0.4]  (0.11, 0.21]  (0.07, 0.11]  ＜0.07 |
