# Supplementary figures and images for: Pollen grading prediction scale for patients with Artemisia pollen allergy in China: A 3‐day moving predictive model
Source: Clin Transl Allergy. 2023 Jul 10;13(7):e12280. doi: 10.1002/clt2.12280 (PMC10332133; doi:10.1002/clt2.12280)

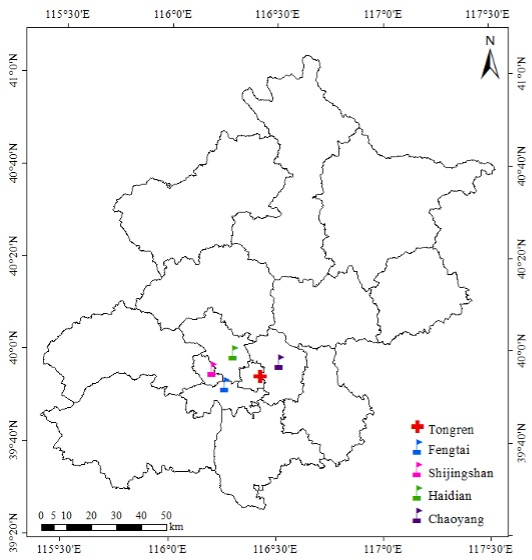

Supplement: Supplementary file 2 — Figure S1 [file CLT2-13-e12280-s005.tiff]

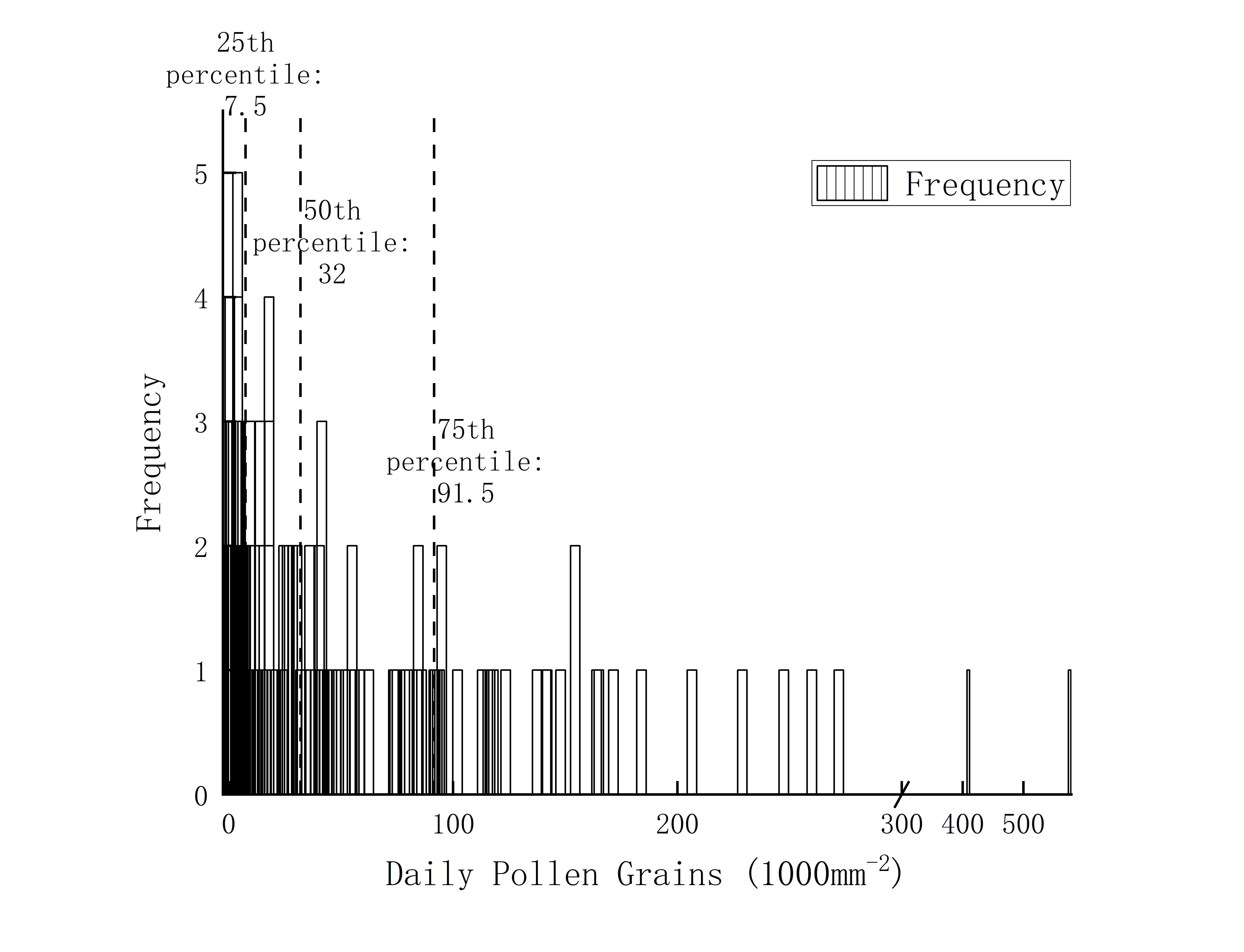

Supplement: Supplementary file 3 — Figure S2 [file CLT2-13-e12280-s007.tif]

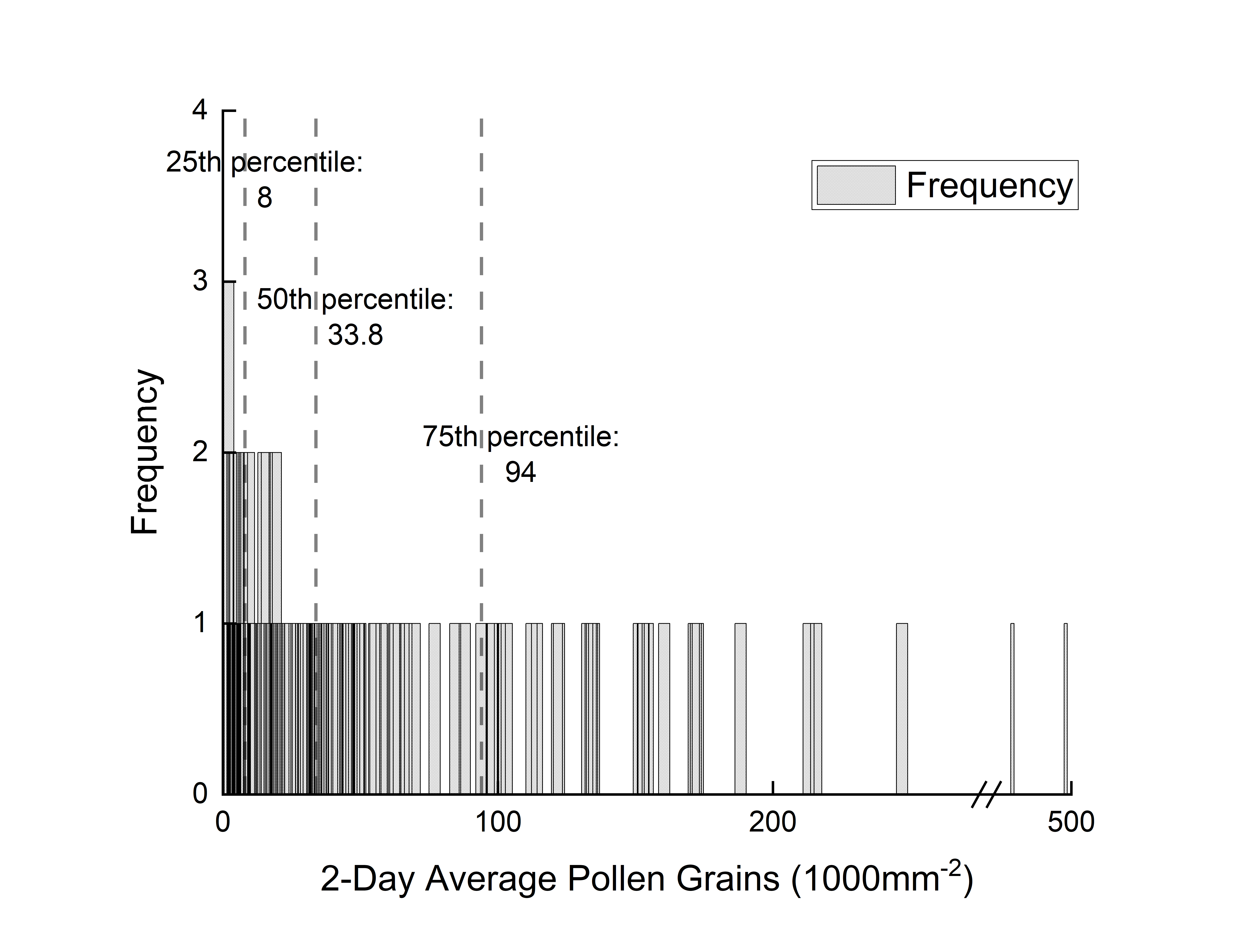

Supplement: Supplementary file 4 — Figure S3 [file CLT2-13-e12280-s004.tif]

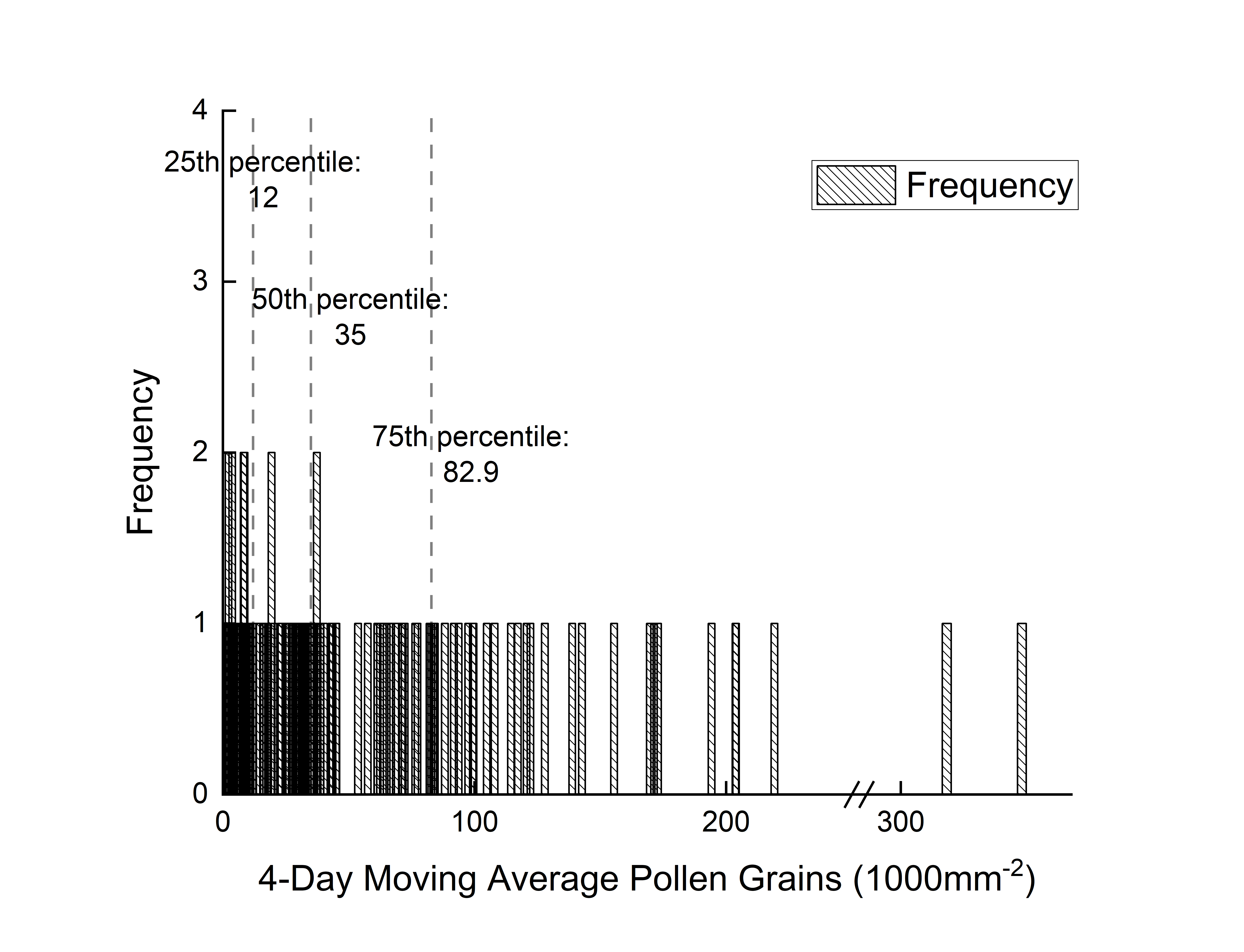

Supplement: Supplementary file 5 — Figure S4 [file CLT2-13-e12280-s003.tif]

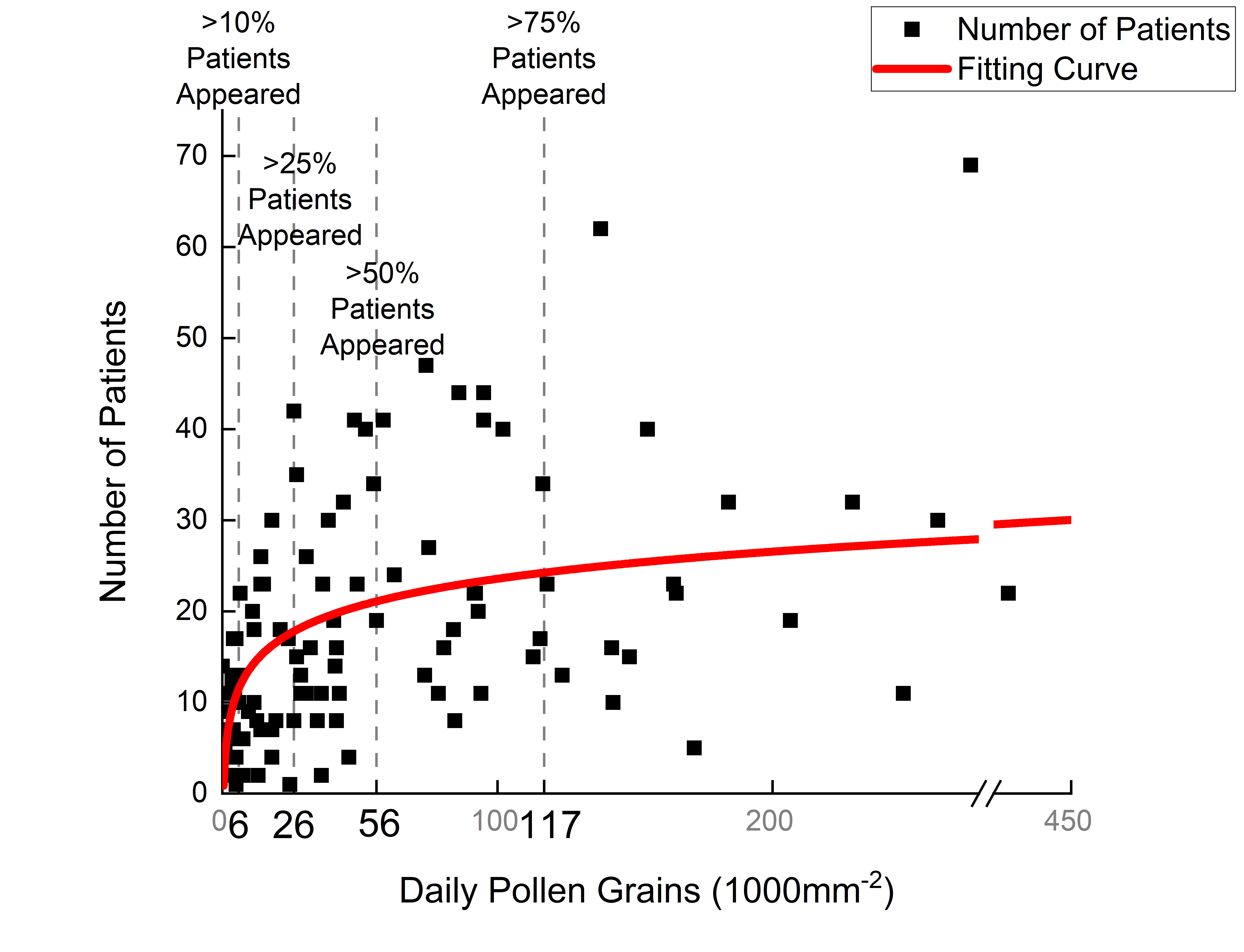

Supplement: Supplementary file 6 — Figure S5 [file CLT2-13-e12280-s006.tif]

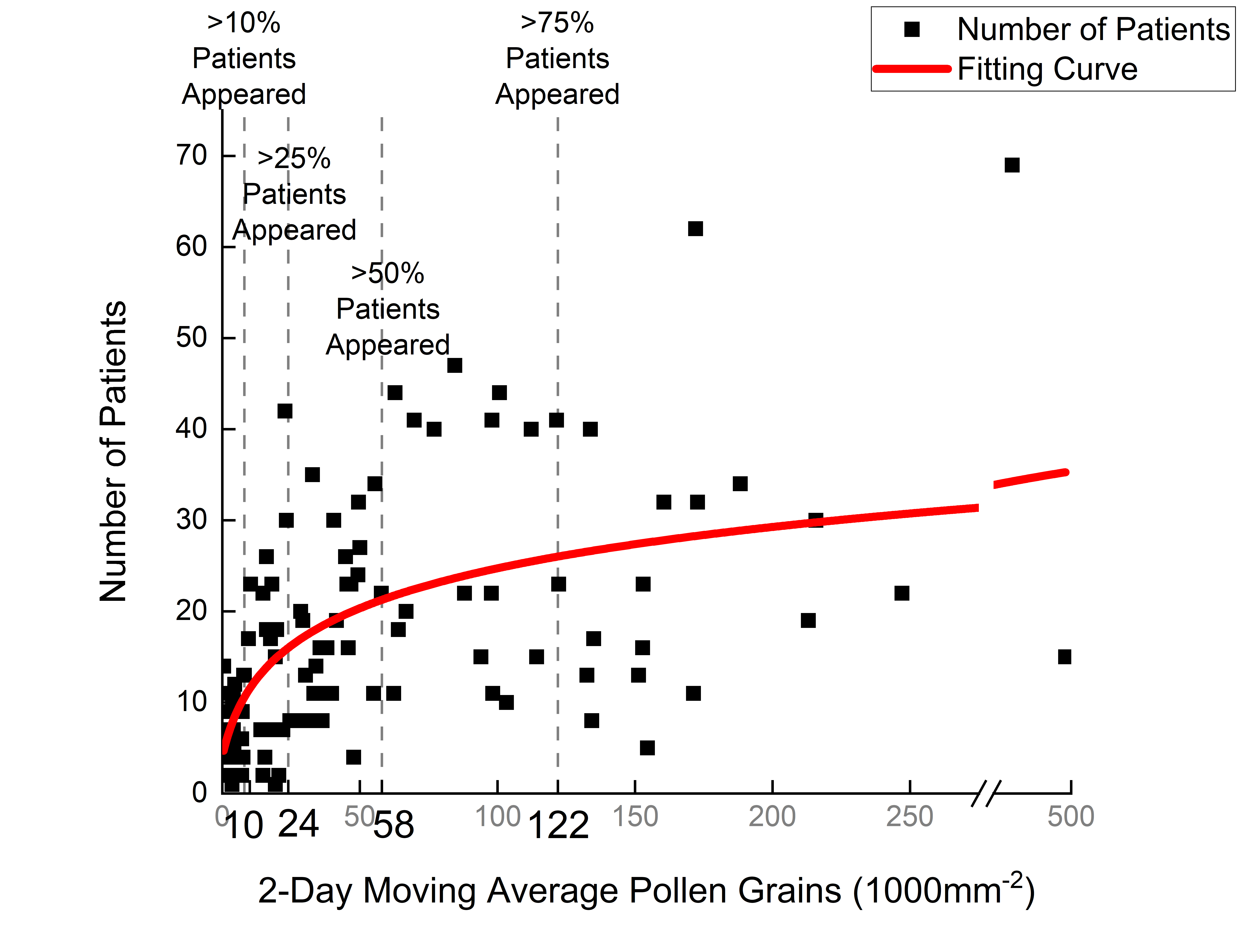

Supplement: Supplementary file 7 — Figure S6 [file CLT2-13-e12280-s001.tif]

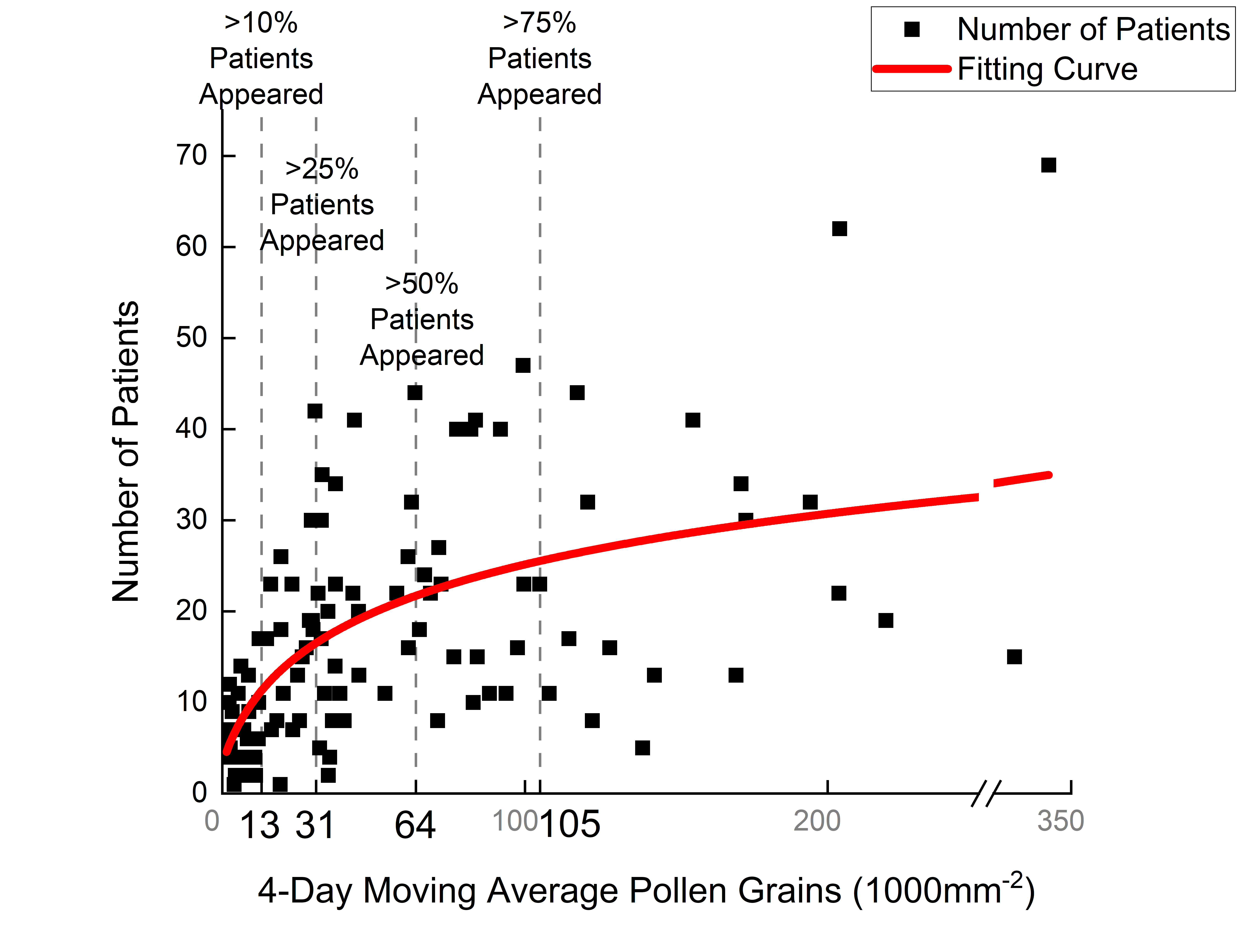

Supplement: Supplementary file 8 — Figure S7 [file CLT2-13-e12280-s002.tif]
